# Crystal structure of a 1:1 cocrystal of nicotinamide with 2-chloro-5-nitro­benzoic acid

**DOI:** 10.1107/S2056989019013859

**Published:** 2019-10-22

**Authors:** Keshab M. Bairagi, Priyanka Pal, Subhrajyoti Bhandary, Katharigatta N. Venugopala, Deepak Chopra, Susanta K. Nayak

**Affiliations:** a Department of Chemistry, Visvesvaraya National Institute of Technology, Nagpur 440 010, Maharashtra, India; b Department of Chemistry, Indian Institute of Science Education and Research Bhopal, Bhauri, Bhopal 462 066, Madhya Pradesh, India; c Department of Pharmaceutical Sciences, College of Clinical Pharmacy, King Faisal University, Al-Ahsa 31982, Kingdom of Saudi Arabia; d Department of Biotechnology and Food Technology, Durban University of Technology, Durban 4001, South Africa

**Keywords:** crystal structure, cocrystal, Hirshfeld surface, 2-chloro-5-nitro­benzoic acid, nicotinamide

## Abstract

In the title 1:1 cocrystal, C_7_H_4_ClNO_4_·C_6_H_6_N_2_O, nicotinamide (NIC) and 2-chloro-5-nitro­benzoic acid (CNBA) cocrystallize with one mol­ecule each of NIC and CNBA in the asymmetric unit. In this structure, CNBA and NIC form hydrogen bonds through O—H⋯N, N—H⋯O and C—H⋯O inter­actions along with N—H⋯O dimer hydrogen bonds of NIC. Further additional weak π–π inter­actions stabilize the mol­ecular assembly of this cocrystal.

## Abstract 2

In the 1:1 cocrystal of nicotinamide and 2-chloro-5-nitro­benzoic acid, the mol­ecules form hydrogen bonds through O—H⋯N, N—H⋯O, and C—H⋯O inter­actions along with N—H⋯O dimer hydrogen bonds of nicotinamide. Further additional weak π–π inter­actions stabilize the mol­ecular assembly of this cocrystal.

## Chemical context   

Nicotinamide (NIC) derivatives are used in various applications, for example, in the prevention of type 1 diabetes (Elliott *et al.*, 1993 ▸) and nicotinamide cofactors are also used in preparative enzymatic synthesis (Chenault & Whitesides, 1987 ▸). The nicotinamide formulation has also been used for treatment in palliative radiotherapy (Horsman *et al.*, 1993 ▸). The pharmacological result for the active pharmaceutical ingredient (API) will increase if it becomes cocrystallized with a coformer or other active com­ponent (Schultheiss & Newman, 2009 ▸; Lemmerer *et al.*, 2010 ▸). Chloro­benzoic acid derivatives are widely used in the pharmaceutical industry. 2-Chloro-4-nitro­benzoic acid is used for immunodeficiency diseases as an anti­viral and anti­cancer agent (Lemmerer *et al.*, 2010 ▸). In the title com­pound, NIC is cocrystallized with the CNBA coformer as it acts as an excellent candidate for cocrystallization because of the hydrogen-bond acceptor and donor parts (Dragovic *et al.*, 1995 ▸).

## Structural commentary   

**Figure 1:**
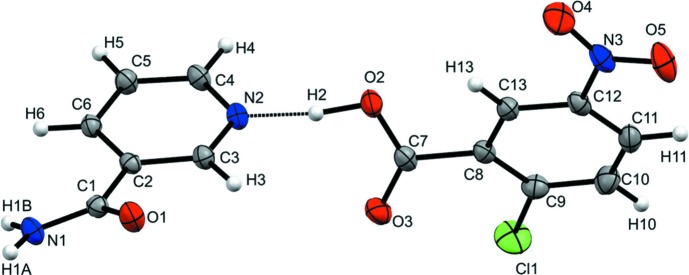
The asymmetric unit of the title com­pound, showing 50% probability ellipsoids, the atom labelling and hydrogen bonding with dotted lines.

The title com­pound CNBA–NIC (1:1) crystallizes in the monoclinic space group *P*2_1_/*c* with four mol­ecules of NIC and CNBA in the unit cell. The dihedral angle between the amide plane with the mean plane of the phenyl part in NIC is 23.87 (1)°, and the dihedral angles of the carboxyl and nitro groups with the chloro­phenyl ring in CNBA are 24.92 (1) and 3.56 (1)°, respectively. In the asymmetric unit, an (CNBA)O–H⋯N inter­action plays a prime role in the mol­ecular recognition of this cocrystal (Fig. 1 ▸).

## Supra­molecular features   

**Figure 2:**
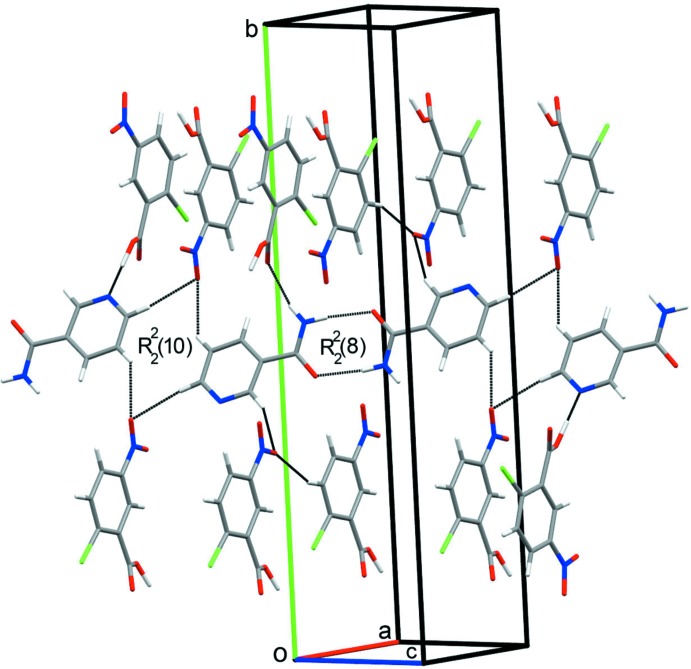
Hydrogen bonds in the title com­pound showing the dimer formation through N—H⋯O inter­actions and tetra­mer formation through C—H⋯O inter­actions.

**Figure 3:**
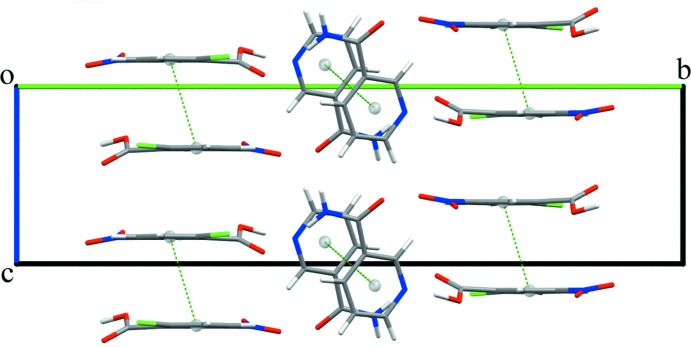
Weak π–π inter­actions stabilize the mol­ecular assembly of both mol­ecules in the crystal.

In the crystal structure of the title cocrystal, a strong (CNBA)O—H⋯N(NIC) hydrogen bond and additional (NIC)N—H⋯O(CNBA) and (NIC)C—H⋯O(CNBA) hydrogen bonds are observed (Fig. 2 ▸ and Table 1 ▸). In this cocrystal, the NIC mol­ecule forms a dimer with itself having an 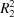
(8) graph-set motif (Etter *et al.*, 1990 ▸). These dimers are further connected *via* C—H⋯O hydrogen bonding and form a tetra­meric ring with two mol­ecules each of NIC and CNBA with 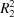
(10) graph-set motifs (Etter *et al.*, 1990 ▸) (Fig. 2 ▸). Furthermore, weak π–π inter­actions are observed for both NIC [3.68 (7) Å] and CNBA [3.73 (7) Å] which stabilize the mol­ecular assembly along the *bc* plane (Fig. 3 ▸).
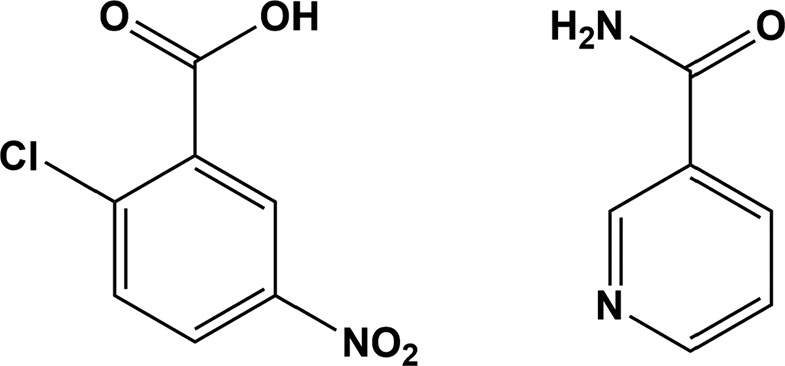


## Hirshfeld surface analysis   

**Figure 4:**
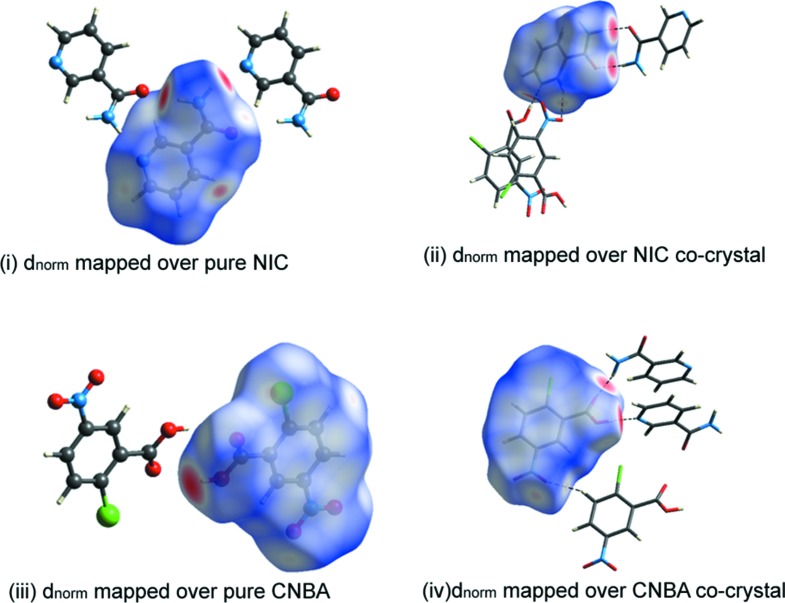
Hirshfeld surfaces developed on (i) *d*_norm_ mapped over the pure NIC mol­ecule, (ii) *d*_norm_ mapped over the NIC mol­ecule in title com­pound, (iii) *d*_norm_ mapped over the pure CNBA mol­ecule and (iv) *d*_norm_ mapped over the CNBA mol­ecule in title com­pound.

**Figure 5:**
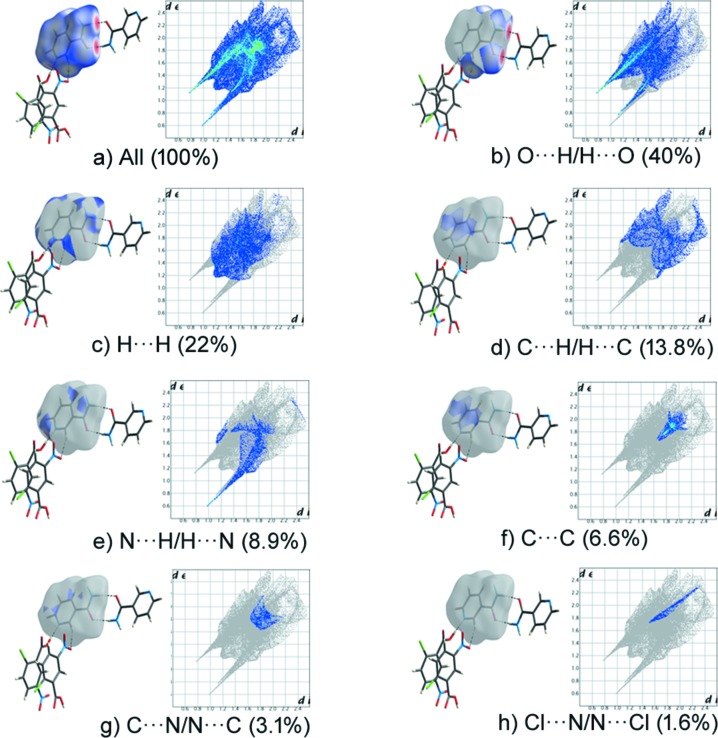
Two-dimensional fingerprint plots and relative contributions of various inter­actions to the Hirshfeld surface of the NIC cocrystal mol­ecule.

**Figure 6:**
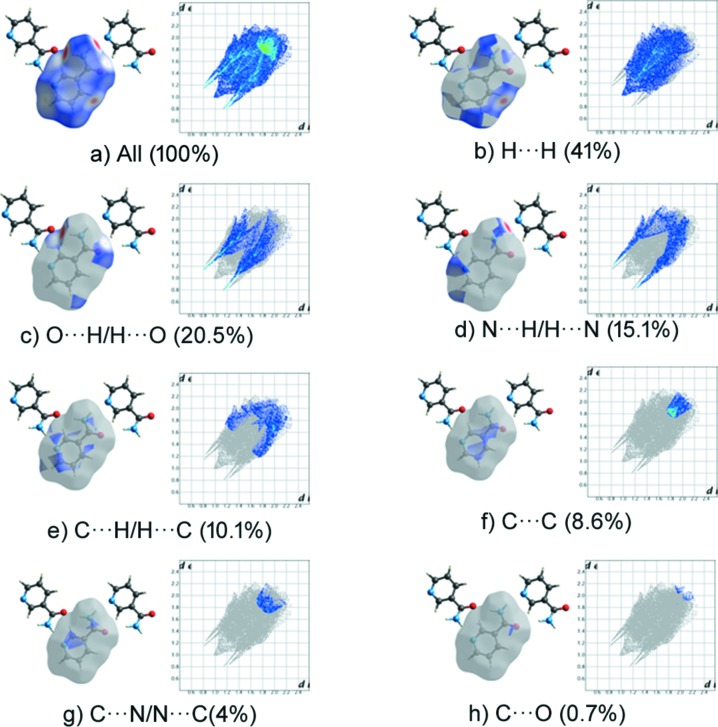
Two-dimensional fingerprint plots and relative contributions of various inter­actions to the Hirshfeld surface of the pure NIC mol­ecule.

**Figure 7:**
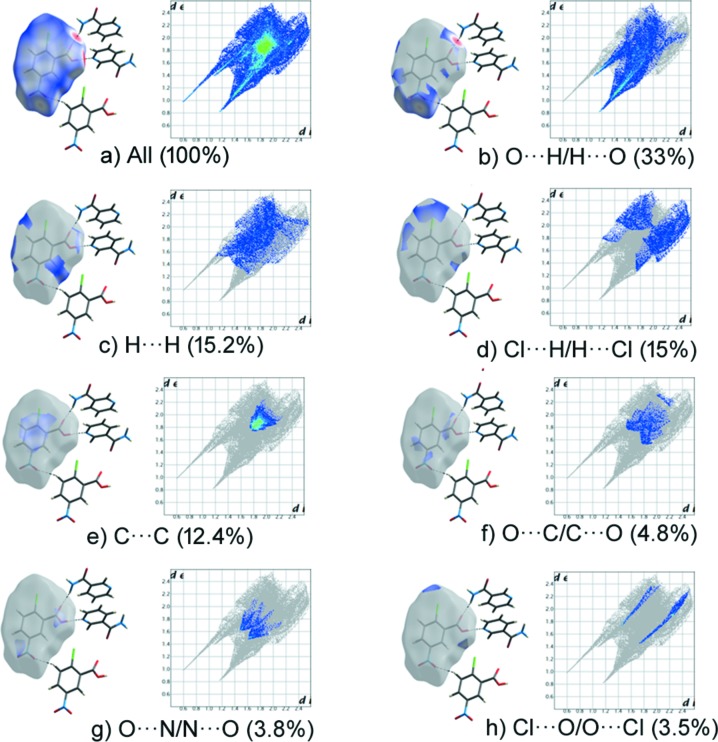
Two-dimensional fingerprint plots and relative contributions of various inter­actions to the Hirshfeld surface of the CNBA cocrystal mol­ecule.

**Figure 8:**
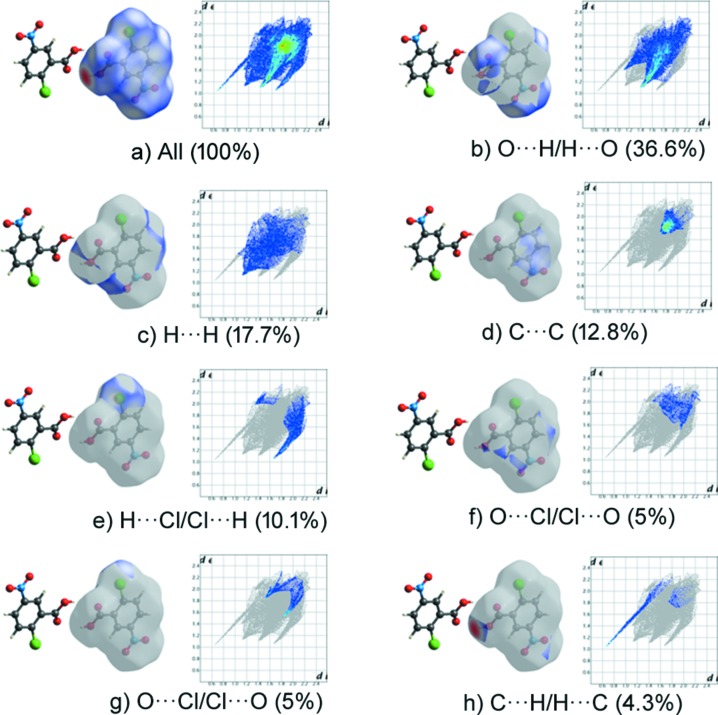
Two-dimensional fingerprint plots and relative contributions of various inter­actions to the Hirshfeld surface of the pure CNBA mol­ecule

To understand the role of inter­molecular inter­actions, we have utilized the Hirshfeld surface analysis visualizing tool (Spackman & Jayatilaka, 2009 ▸). The Hirshfeld surfaces and two-dimensional fingerprint plots developed using *CrystalExplorer* (Version 3.1; Wolff *et al.*, 2012 ▸) are shown in Fig. 4 ▸. The red spot on the surface represents a strong inter­action through O—H⋯N and N—H⋯O hydrogen bonding, whereas the blue color represents a lack of inter­action. The *d*_norm_ map of the title com­pound NIC·CNBA and its pure com­ponents is shown in Fig. 4 ▸, where individual mol­ecular inter­actions were estimated. The fingerprint plot shows that O⋯H/H⋯O and H⋯H contribute the major part of the inter­action in all com­pounds (Fig. 4 ▸). The O⋯H/H⋯O contact contributes 40% to the cocrystal NIC mol­ecule (Fig. 5 ▸) and 20.5% to the pure NIC mol­ecule (NICOAM01; Miwa *et al.*, 1999 ▸) (Fig. 6 ▸), and H⋯H contributes 22% to the cocrystal NIC mol­ecule and 41% to the pure NIC mol­ecule. Similarly, O⋯H/H⋯O contacts contribute 33% to the cocrystal CNBA mol­ecule (Fig. 7 ▸) and 36.6% to the pure CNBA mol­ecule (CLNBZA; Ferguson & Sim, 1962 ▸) (Fig. 8 ▸), and H⋯H contributes 15.2% to the cocrystal CNBA mol­ecule and 17.7% to the pure NIC mol­ecule.

## Database survey   

A search for the title cocrystal in the Cambridge Structural Database (CSD, Version 5.40, update of February 2019; Groom *et al.*, 2016 ▸) found no hits. However, searches for NIC and CNBA gave 237 and 9 hits, respectively. A search for the NIC mol­ecule showed that the N atom on the phenyl ring forms strong O—H⋯N hydrogen bonds with a carboxyl H atom in the most of the cocrystals [ABULIU (Lou & Hu, 2011 ▸), BICQAH (Aitipamula *et al.*, 2013 ▸), BICQEL (Aitipamula *et al.*, 2013 ▸), BOBQUG (Zhang *et al.*, 2013 ▸), CUYXUQ (Lemmerer & Bernstein, 2010 ▸), DINRUP (Lemmerer *et al.*, 2013 ▸), DINSEA (Lemmerer *et al.*, 2013 ▸), EDAPOQ (Orola & Veidis, 2009 ▸) *etc*]. For the CNBA search, two structures were found similar to the title com­pound where strong hydrogen bonding is formed by the carboxyl H atom with a pyridine N atom [AJIWIA (Gotoh & Ishida, 2009 ▸) and OCAZAT (Ishida *et al.*, 2001 ▸)]. AJIWIA also shows halogen bonds through C—O⋯Cl bonding and forms a dimer through C—H⋯O hydrogen bonding.

## Synthesis and crystallization   

All the chemicals used for the synthesis were purchased from Alfa Aesar and used without further purification. A stock solution was prepared from an equimolar mixture of 2-chloro-5 nitro­benzoic acid (82.44 mg, 0.409 mmol) and nicotinamide (50 mg, 0.409 mmol) in a minimum amount of ethanol and made up to a volume of 10 ml. Ten different combinations of the mixture were prepared using ethanol–hexane as the solvent mixture over the ratio range 1:1 to 1:10. The mixture was kept in a 5 ml beaker and covered with parafilm, with four to five small holes in it. These solutions were allowed to evaporate slowly at room temperature (27 °C) over several days to obtain single crystals. After a few days, colourless crystals were obtained from ethanol–hexane solutions with concentration ratios of 1:10, 1:2 and 1:4. The melting point of the obtained crystal was 159.7 °C.

## Refinement   

Crystal data, data collection, and structure refinement details are summarized in Table 2 ▸. All H atoms were found in a difference Fourier maps and were refind freely.

## Supplementary Material

Crystal structure: contains datablock(s) I, global. DOI: 10.1107/S2056989019013859/eb2025sup1.cif

Structure factors: contains datablock(s) I. DOI: 10.1107/S2056989019013859/eb2025Isup2.hkl

CCDC references: 1958621, 1958621

Additional supporting information:  crystallographic information; 3D view; checkCIF report

## Figures and Tables

**Table 1:** Hydrogen-bond geometry (Å, °)

*D*—H⋯*A*	*D*—H	H⋯*A*	*D*⋯*A*	*D*—H⋯*A*
N1—H1*A*⋯O1	0.90 (2)	2.005 (19)	2.9004 (15)	171.4 (14)
N1—H1*B*⋯O3	0.873 (19)	2.116 (19)	2.9715 (14)	166.6 (16)
O2—H2⋯N2	1.07 (2)	1.49 (2)	2.5543 (13)	172 (2)
C3—H3⋯O4	0.986 (15)	2.516 (15)	3.4505 (16)	158.2 (12)
C4—H4⋯O5	0.979 (14)	2.442 (15)	3.3256 (17)	149.9 (12)
C5—H5⋯O5	0.956 (15)	2.450 (16)	3.0878 (17)	124.0 (11)
C6—H6⋯O3	0.959 (15)	2.608 (15)	3.452 (1)	147.0 (11)
C10—H10⋯O4	0.955 (15)	2.599 (16)	3.5010 (18)	157.6 (15)

**Table 2:** Experimental details

Crystal data	
Chemical formula	C_7_H_4_ClNO_4_·C_6_H_6_N_2_O
*M*_r_	323.69
Crystal system, space group	Monoclinic, *P*2_1_/*c*
Temperature (K)	123
*a*, *b*, *c* (Å)	7.4897 (1), 26.3607 (5), 7.0623 (1)
β (°)	96.356 (1)
*V* (Å^3^)	1385.77 (4)
*Z*	4
Radiation type	Mo *K*α
μ (mm^−1^)	0.31
Crystal size (mm)	0.28 × 0.22 × 0.15

Data collection	
Diffractometer	Bruker Kappa APEXII DUO
Absorption correction	Multi-scan (*SADABS*; Bruker, 2001 ▸)
*T*_min_, *T*_max_	0.874, 0.908
No. of measured, independent and observed [*I* > 2σ(*I*)] reflections	29950, 4123, 3316
*R*_int_	0.037
(sin θ/λ)_max_ (Å^−1^)	0.708

Refinement	
*R*[*F* ^2^ > 2σ(*F* ^2^)], *wR*(*F* ^2^), *S*	0.038, 0.094, 1.08
No. of reflections	4123
No. of parameters	239
H-atom treatment	All H-atom parameters refined
Δρ_max_, Δρ_min_ (e Å^−3^)	0.30, −0.26

Computer programs: *APEX2* (Bruker, 2012 ▸), *SAINT* (Bruker, 2012 ▸), *SHELXS18* (Sheldrick, 2015 ▸), *SHELXL2018* (Sheldrick, 2015 ▸), *Mercury* (Macrae *et al.*, 2008 ▸), *OLEX2* (Dolomanov *et al.*, 2009 ▸) and *PLATON* (Spek, 2009 ▸).

## supplementary crystallographic information

### Crystal data

**Table:**

C_7_H_4_ClNO_4_·C_6_H_6_N_2_O	*D*_x_ = 1.551 Mg m^−^^3^
*M**_r_* = 323.69	Melting point: 159.7 K
Monoclinic, *P*2_1_/*c*	Mo *K*α radiation, λ = 0.71073 Å
*a* = 7.4897 (1) Å	Cell parameters from 4123 reflections
*b* = 26.3607 (5) Å	θ = 1.5–30.2°
*c* = 7.0623 (1) Å	µ = 0.31 mm^−^^1^
β = 96.356 (1)°	*T* = 123 K
*V* = 1385.77 (4) Å^3^	Block, colorless
*Z* = 4	0.28 × 0.22 × 0.15 mm
*F*(000) = 664	

### Data collection

**Table:**

Bruker Kappa APEXII DUO diffractometer	4123 independent reflections
Radiation source: fine-focus sealed tube	3316 reflections with *I* > 2σ(*I*)
Graphite monochromator	*R*_int_ = 0.037
ω scans	θ_max_ = 30.2°, θ_min_ = 1.6°
Absorption correction: multi-scan (SADABS; Bruker, 2001)	*h* = −10→10
*T*_min_ = 0.874, *T*_max_ = 0.908	*k* = −37→37
29950 measured reflections	*l* = −9→9

### Refinement

**Table:**

Refinement on *F*^2^	0 restraints
Least-squares matrix: full	Hydrogen site location: difference Fourier map
*R*[*F*^2^ > 2σ(*F*^2^)] = 0.038	All H-atom parameters refined
*wR*(*F*^2^) = 0.094	*w* = 1/[σ^2^(*F*_o_^2^) + (0.0394*P*)^2^ + 0.4303*P*] where *P* = (*F*_o_^2^ + 2*F*_c_^2^)/3
*S* = 1.08	(Δ/σ)_max_ < 0.001
4123 reflections	Δρ_max_ = 0.30 e Å^−^^3^
239 parameters	Δρ_min_ = −0.26 e Å^−^^3^

### Special details

**Table:**

Geometry. All esds (except the esd in the dihedral angle between two l.s. planes) are estimated using the full covariance matrix. The cell esds are taken into account individually in the estimation of esds in distances, angles and torsion angles; correlations between esds in cell parameters are only used when they are defined by crystal symmetry. An approximate (isotropic) treatment of cell esds is used for estimating esds involving l.s. planes.
Refinement. Single-crystal X-ray diffraction data were collected on a Bruker KAPPA APEX II DUO diffractometer using graphite-monochromated Mo-Kα radiation (λ = 0.71073Å)(Bruker, 2012). The data collection was performed at 153 (2) K. The temperature was monitored by an Oxford Cryostream cooling system (Oxford Cryostat). the program SAINT (Bruker, 2012) were used for cell refinement and data reduction. The data were scaled and absorption correction performed using SADABS(Bruker, 2001). The structure was solved by direct methods using SHELXS-18(Sheldrick, 2015) and refined by full-matrix least-squares methods based on F2 using SHELXL-2018/3(Sheldrick, 2015). The computing , Mercury(Macrae *et al.*, 2008) and PLATON (Spek, 2009) were used for molecular graphics and molecular interactions. All non-hydrogen atoms were refined anisotropically.

### Fractional atomic coordinates and isotropic or equivalent isotropic displacement parameters (Å^2^)

**Table:**

	*x*	*y*	*z*	*U*_iso_*/*U*_eq_	
Cl1	−0.18141 (5)	0.18331 (2)	0.33099 (6)	0.04137 (12)	
O2	0.39368 (12)	0.16413 (3)	0.29015 (14)	0.0267 (2)	
O1	0.87918 (13)	0.45197 (3)	1.35865 (13)	0.0268 (2)	
O5	0.29333 (16)	0.39338 (4)	0.38136 (16)	0.0381 (3)	
O3	0.16833 (14)	0.13103 (3)	0.43221 (14)	0.0308 (2)	
O4	0.50015 (15)	0.34087 (4)	0.32242 (18)	0.0417 (3)	
N1	0.87836 (15)	0.53606 (4)	1.29452 (16)	0.0226 (2)	
N2	0.57395 (14)	0.41744 (4)	0.85619 (15)	0.0208 (2)	
N3	0.34615 (16)	0.35059 (4)	0.35149 (16)	0.0262 (2)	
C6	0.75383 (15)	0.50921 (4)	0.89924 (17)	0.0189 (2)	
C1	0.83945 (15)	0.48801 (4)	1.24921 (17)	0.0193 (2)	
C2	0.74506 (15)	0.47734 (4)	1.05512 (16)	0.0172 (2)	
C12	0.21719 (17)	0.30889 (4)	0.35036 (17)	0.0209 (2)	
C4	0.59051 (17)	0.44720 (5)	0.70484 (18)	0.0221 (2)	
C13	0.28144 (16)	0.25993 (4)	0.35032 (17)	0.0193 (2)	
C5	0.67736 (17)	0.49327 (5)	0.72106 (18)	0.0217 (2)	
C9	−0.02066 (17)	0.23039 (5)	0.34499 (19)	0.0243 (3)	
C3	0.65244 (16)	0.43172 (4)	1.02733 (17)	0.0201 (2)	
C8	0.16239 (16)	0.21927 (4)	0.35036 (16)	0.0191 (2)	
C10	−0.08225 (18)	0.28024 (5)	0.3432 (2)	0.0291 (3)	
C7	0.24078 (17)	0.16659 (4)	0.36051 (17)	0.0205 (2)	
C11	0.03681 (19)	0.32024 (5)	0.34746 (19)	0.0261 (3)	
H3	0.639 (2)	0.4084 (6)	1.134 (2)	0.022 (4)*	
H6	0.813 (2)	0.5415 (6)	0.909 (2)	0.022 (4)*	
H13	0.404 (2)	0.2537 (6)	0.352 (2)	0.028 (4)*	
H4	0.540 (2)	0.4339 (6)	0.581 (2)	0.025 (4)*	
H5	0.691 (2)	0.5138 (6)	0.612 (2)	0.026 (4)*	
H1A	0.947 (2)	0.5426 (6)	1.405 (3)	0.035 (4)*	
H1B	0.850 (2)	0.5613 (7)	1.216 (3)	0.038 (5)*	
H11	−0.006 (2)	0.3550 (7)	0.346 (3)	0.040 (5)*	
H10	−0.208 (3)	0.2870 (7)	0.338 (3)	0.042 (5)*	
H2	0.462 (3)	0.1292 (9)	0.326 (3)	0.074 (7)*	

### Atomic displacement parameters (Å^2^)

**Table:**

	*U*^11^	*U*^22^	*U*^33^	*U*^12^	*U*^13^	*U*^23^
Cl1	0.02685 (18)	0.0403 (2)	0.0562 (3)	−0.01523 (14)	0.00135 (15)	0.00272 (16)
O2	0.0251 (5)	0.0190 (4)	0.0362 (5)	0.0037 (3)	0.0040 (4)	0.0050 (4)
O1	0.0362 (5)	0.0207 (4)	0.0216 (5)	−0.0011 (4)	−0.0050 (4)	0.0024 (3)
O5	0.0529 (7)	0.0147 (4)	0.0430 (6)	−0.0008 (4)	−0.0109 (5)	−0.0029 (4)
O3	0.0424 (6)	0.0172 (4)	0.0346 (5)	−0.0037 (4)	0.0115 (4)	0.0017 (4)
O4	0.0345 (6)	0.0268 (5)	0.0654 (8)	−0.0094 (4)	0.0126 (5)	0.0039 (5)
N1	0.0265 (5)	0.0186 (5)	0.0213 (5)	0.0006 (4)	−0.0040 (4)	−0.0017 (4)
N2	0.0211 (5)	0.0163 (5)	0.0243 (5)	−0.0016 (4)	−0.0008 (4)	−0.0014 (4)
N3	0.0357 (6)	0.0165 (5)	0.0253 (6)	−0.0031 (4)	−0.0022 (4)	0.0021 (4)
C6	0.0196 (5)	0.0151 (5)	0.0220 (6)	−0.0004 (4)	0.0022 (4)	−0.0009 (4)
C1	0.0187 (5)	0.0192 (6)	0.0200 (6)	0.0007 (4)	0.0023 (4)	−0.0015 (4)
C2	0.0167 (5)	0.0164 (5)	0.0184 (5)	0.0018 (4)	0.0015 (4)	−0.0014 (4)
C12	0.0275 (6)	0.0164 (5)	0.0184 (6)	−0.0018 (4)	0.0006 (4)	0.0000 (4)
C4	0.0247 (6)	0.0197 (6)	0.0207 (6)	0.0010 (5)	−0.0022 (5)	−0.0025 (4)
C13	0.0209 (5)	0.0183 (5)	0.0186 (6)	−0.0003 (4)	0.0011 (4)	0.0004 (4)
C5	0.0268 (6)	0.0184 (6)	0.0194 (6)	0.0004 (4)	0.0006 (5)	0.0017 (4)
C9	0.0216 (6)	0.0256 (6)	0.0257 (6)	−0.0044 (5)	0.0025 (5)	0.0000 (5)
C3	0.0216 (5)	0.0173 (5)	0.0212 (6)	−0.0002 (4)	0.0015 (4)	0.0001 (4)
C8	0.0216 (5)	0.0177 (5)	0.0177 (6)	−0.0017 (4)	0.0012 (4)	−0.0002 (4)
C10	0.0217 (6)	0.0320 (7)	0.0335 (7)	0.0045 (5)	0.0027 (5)	0.0004 (5)
C7	0.0266 (6)	0.0167 (5)	0.0174 (6)	−0.0019 (4)	−0.0011 (4)	−0.0008 (4)
C11	0.0305 (7)	0.0218 (6)	0.0255 (7)	0.0061 (5)	0.0010 (5)	−0.0004 (5)

### Geometric parameters (Å, º)

**Table:**

Cl1—C9	1.7244 (13)	C1—C2	1.4977 (16)
O2—C7	1.2994 (15)	C2—C3	1.3913 (16)
O2—H2	1.07 (2)	C12—C13	1.3774 (16)
O1—C1	1.2399 (14)	C12—C11	1.3816 (18)
O5—N3	1.2215 (15)	C4—C5	1.3766 (17)
O3—C7	1.2208 (14)	C4—H4	0.977 (16)
O4—N3	1.2209 (16)	C13—C8	1.3941 (16)
N1—C1	1.3307 (16)	C13—H13	0.932 (16)
N1—H1A	0.901 (19)	C5—H5	0.957 (16)
N1—H1B	0.876 (18)	C9—C10	1.3921 (19)
N2—C3	1.3383 (16)	C9—C8	1.3984 (17)
N2—C4	1.3426 (16)	C3—H3	0.984 (15)
N3—C12	1.4626 (16)	C8—C7	1.5064 (16)
C6—C5	1.3887 (17)	C10—C11	1.379 (2)
C6—C2	1.3922 (16)	C10—H10	0.955 (18)
C6—H6	0.959 (15)	C11—H11	0.971 (18)
			
C7—O2—H2	111.9 (13)	C12—C13—H13	120.6 (9)
C1—N1—H1A	118.6 (11)	C8—C13—H13	119.6 (9)
C1—N1—H1B	122.7 (12)	C4—C5—C6	119.08 (11)
H1A—N1—H1B	118.4 (16)	C4—C5—H5	121.5 (9)
C3—N2—C4	118.98 (10)	C6—C5—H5	119.4 (9)
O4—N3—O5	123.56 (12)	C10—C9—C8	121.40 (11)
O4—N3—C12	118.49 (11)	C10—C9—Cl1	116.76 (10)
O5—N3—C12	117.95 (12)	C8—C9—Cl1	121.80 (10)
C5—C6—C2	118.89 (11)	N2—C3—C2	122.20 (11)
C5—C6—H6	118.5 (9)	N2—C3—H3	116.1 (9)
C2—C6—H6	122.6 (9)	C2—C3—H3	121.7 (9)
O1—C1—N1	123.25 (11)	C13—C8—C9	117.65 (11)
O1—C1—C2	118.88 (10)	C13—C8—C7	117.57 (10)
N1—C1—C2	117.87 (11)	C9—C8—C7	124.77 (11)
C3—C2—C6	118.46 (11)	C11—C10—C9	120.57 (12)
C3—C2—C1	117.96 (10)	C11—C10—H10	119.3 (11)
C6—C2—C1	123.47 (10)	C9—C10—H10	120.1 (11)
C13—C12—C11	122.95 (11)	O3—C7—O2	124.84 (11)
C13—C12—N3	118.27 (11)	O3—C7—C8	122.60 (11)
C11—C12—N3	118.78 (11)	O2—C7—C8	112.54 (10)
N2—C4—C5	122.26 (11)	C10—C11—C12	117.62 (12)
N2—C4—H4	116.2 (9)	C10—C11—H11	120.6 (11)
C5—C4—H4	121.6 (9)	C12—C11—H11	121.8 (11)
C12—C13—C8	119.79 (11)		
			
C5—C6—C2—C3	−2.96 (17)	C1—C2—C3—N2	−175.60 (10)
C5—C6—C2—C1	173.20 (11)	C12—C13—C8—C9	1.77 (17)
O1—C1—C2—C3	21.57 (16)	C12—C13—C8—C7	−176.93 (11)
N1—C1—C2—C3	−159.19 (11)	C10—C9—C8—C13	−1.15 (19)
O1—C1—C2—C6	−154.61 (12)	Cl1—C9—C8—C13	176.29 (9)
N1—C1—C2—C6	24.63 (17)	C10—C9—C8—C7	177.44 (12)
O4—N3—C12—C13	11.29 (17)	Cl1—C9—C8—C7	−5.12 (18)
O5—N3—C12—C13	−168.81 (12)	C8—C9—C10—C11	−0.3 (2)
O4—N3—C12—C11	−168.07 (12)	Cl1—C9—C10—C11	−177.83 (11)
O5—N3—C12—C11	11.83 (17)	C13—C8—C7—O3	151.23 (12)
C3—N2—C4—C5	−3.55 (18)	C9—C8—C7—O3	−27.36 (19)
C11—C12—C13—C8	−1.03 (19)	C13—C8—C7—O2	−26.97 (15)
N3—C12—C13—C8	179.64 (11)	C9—C8—C7—O2	154.44 (12)
N2—C4—C5—C6	1.33 (19)	C9—C10—C11—C12	1.0 (2)
C2—C6—C5—C4	1.97 (17)	C13—C12—C11—C10	−0.4 (2)
C4—N2—C3—C2	2.47 (17)	N3—C12—C11—C10	178.92 (12)
C6—C2—C3—N2	0.78 (17)		

### Hydrogen-bond geometry (Å, º)

**Table:**

*D*—H···*A*	*D*—H	H···*A*	*D*···*A*	*D*—H···*A*
N1—H1*A*···O1	0.90 (2)	2.005 (19)	2.9004 (15)	171.4 (14)
N1—H1*B*···O3	0.873 (19)	2.116 (19)	2.9715 (14)	166.6 (16)
O2—H2···N2	1.07 (2)	1.49 (2)	2.5543 (13)	172 (2)
C3—H3···O4	0.986 (15)	2.516 (15)	3.4505 (16)	158.2 (12)
C4—H4···O5	0.979 (14)	2.442 (15)	3.3256 (17)	149.9 (12)
C5—H5···O5	0.956 (15)	2.450 (16)	3.0878 (17)	124.0 (11)
C6—H6···O3	0.959 (15)	2.608 (15)	3.452 (1)	147.0 (11)
C10—H10···O4	0.955 (15)	2.599 (16)	3.5010 (18)	157.6 (15)

## References

[bb1] Aitipamula, S., Wong, A. B., Chow, P. S. & Tan, R. B. (2013). *CrystEngComm*, **15**, 5877–5887.

[bb2] Bruker (2001). *SADABS*. Bruker AXS Inc., Madison, Wisconsin, USA.

[bb3] Bruker (2012). *APEX2*. Bruker AXS Inc., Madison, Wisconsin, USA.

[bb4] Chenault, H. K. & Whitesides, G. M. (1987). *Appl. Biochem. Biotechnol.* **14**, 147–197. 10.1007/BF02798431 3304160

[bb5] Dolomanov, O. V., Bourhis, L. J., Gildea, R. J., Howard, J. A. K. & Puschmann, H. (2009). *J. Appl. Cryst.* **42**, 339–341.

[bb6] Dragovic, J., Kim, S. H., Brown, S. L. & Kim, J. H. (1995). *Radiother. Oncol.* **36**, 225–228. 10.1016/0167-8140(95)01581-z 8532910

[bb7] Elliott, R., Pilcher, C., Stewart, A., Fergusson, D. & McGregor, M. (1993). *Ann. N. Y. Acad. Sci.* **696**, 333–341. 10.1111/j.1749-6632.1993.tb17169.x 8109840

[bb8] Etter, M. C., MacDonald, J. C. & Bernstein, J. (1990). *Acta Cryst.* B**46**, 256–262. 10.1107/s0108768189012929 2344397

[bb9] Ferguson, G. & Sim, G. (1962). *J. Chem. Soc.* pp. 1767–1775.

[bb10] Gotoh, K. & Ishida, H. (2009). *Acta Cryst.* C**65**, o534–o538. 10.1107/S0108270109037688 19805889

[bb11] Groom, C. R., Bruno, I. J., Lightfoot, M. P. & Ward, S. C. (2016). *Acta Cryst.* B**72**, 171–179. 10.1107/S2052520616003954 PMC4822653 27048719

[bb12] Horsman, M. R., Hoyer, M., Honess, D. J., Dennis, I. F. & Overgaard, J. (1993). *Radiother. Oncol.* **27**, 131–139. 10.1016/0167-8140(93)90133-s 8356223

[bb13] Ishida, H., Rahman, B. & Kashino, S. (2001). *Acta Cryst.* C**57**, 876–879. 10.1107/s0108270101007016 11443273

[bb14] Lemmerer, A., Adsmond, D. A., Esterhuysen, C. & Bernstein, J. (2013). *Cryst. Growth Des.* **13**, 3935–3952.

[bb15] Lemmerer, A. & Bernstein, J. (2010). *CrystEngComm*, **12**, 2029–2033.

[bb16] Lemmerer, A., Esterhuysen, C. & Bernstein, J. (2010). *J. Pharm. Sci.* **99**, 4054–4071. 10.1002/jps.22211 20574994

[bb17] Lou, B. & Hu, S. (2011). *J. Chem. Crystallogr.* **41**, 1663–1668.

[bb18] Macrae, C. F., Bruno, I. J., Chisholm, J. A., Edgington, P. R., McCabe, P., Pidcock, E., Rodriguez-Monge, L., Taylor, R., van de Streek, J. & Wood, P. A. (2008). *J. Appl. Cryst.* **41**, 466–470.

[bb19] Miwa, Y., Mizuno, T., Tsuchida, K., Taga, T. & Iwata, Y. (1999). *Acta Cryst.* B**55**, 78–84. 10.1107/s0108768198007848 10927341

[bb20] Orola, L. & Veidis, M. V. (2009). *CrystEngComm*, **11**, 415–417.

[bb21] Schultheiss, N. & Newman, A. (2009). *Cryst. Growth Des.* **9**, 2950–2967. 10.1021/cg900129f PMC2690398 19503732

[bb22] Sheldrick, G. M. (2015). *Acta Cryst.* C**71**, 3–8.

[bb23] Spackman, M. A. & Jayatilaka, D. (2009). *CrystEngComm*, **11**, 19–32.

[bb24] Spek, A. L. (2009). *Acta Cryst.* D**65**, 148–155. 10.1107/S090744490804362X PMC2631630 19171970

[bb25] Wolff, S., Grimwood, D., McKinnon, J., Turner, M., Jayatilaka, D. & Spackman, M. (2012). *CrystalExplorer*. The University of Western Australia.

[bb26] Zhang, S.-W., Harasimowicz, M. T., de Villiers, M. M. & Yu, L. (2013). *J. Am. Chem. Soc.* **135**, 18981–18989. 10.1021/ja4103887 24215608

